# Identifying a Marine-Derived Small-Molecule Nucleoprotein Inhibitor Against Influenza A Virus

**DOI:** 10.3390/md23110413

**Published:** 2025-10-23

**Authors:** Zihan Wang, Yang Zhang, Shangjie Xu, Lishan Sun, Hongwei Zhao, Wei Wang

**Affiliations:** Key Laboratory of Marine Drugs, School of Medicine and Pharmacy, Ocean University of China, Chinese Ministry of Education, 5 Yushan Road, Qingdao 266003, China; wzzz0911@163.com (Z.W.); 17630979435@163.com (S.X.); 17806274691@163.com (L.S.); hweizhao2024@163.com (H.Z.)

**Keywords:** influenza A virus, mycophenolic acid methyl ester, nucleoprotein, vRNP assembly

## Abstract

Influenza A virus (IAV) poses a major threat to global public health, exerting immense pressure on human health and the economy. The IAV nucleoprotein (NP) is an ideal target for antiviral drug development. Through Mini-genome and Surface Plasmon Resonance assays, this study discovered and verified that mycophenolic acid methyl ester (MAE), a secondary metabolite produced by the marine algal-associated fungus *Phaeosphaeria spartinae*, can target the viral nucleoprotein to exert anti-IAV activity. Pull-down assays and immunofluorescence have revealed that MAE blocks the nuclear import of viral ribonucleoprotein complexes (vRNP) by interfering with the interaction between NP and IMP-α. It also affects the vRNP assembly process by regulating NP oligomerization and the interaction between NP and PB2. In addition, Sandwich ELISA and Electron Microscopy experiments showed that MAE can also inactivate viral particles to reduce the risk of infection. Comprehensive research results indicate that MAE exerts its effects by inhibiting the viral NP protein, which has laid an important foundation for the development of marine-derived NP-targeted drugs.

## 1. Introduction

Influenza A virus (IAV) is a global public health concern, which causes the seasonal flu and sporadic pandemics, thereby posing a serious threat to human health [1,2,3]. IAV caused at least three large-scale influenza pandemics in last century, the worst of which was the 1918 “Spanish flu,” which caused more than 50 million deaths [4,5]. The seasonal influenza caused by H1N1 and H3N2 virus can cause about 290,000–650,000 deaths per year worldwide [6]. Owing to the high mutation rate of the surface proteins of IAV, current vaccines cannot effectively prevent the infection of newly emerging strains [7,8,9]. Currently, the anti-IAV drugs approved for clinical use mainly target viral envelope proteins such as the neuraminidase and ion-channel inhibitors [10]. However, the emergence of amantadine and oseltamivir resistant IAV strains has led to a decline in the efficacy of these drugs [11]. Although the new antiviral drugs, such as baloxavir, have been approved in recent years, concerns regarding drug resistance and toxicity still remain [12]. Therefore, the development of novel broad-spectrum anti-IAV drugs is urgently needed to address this issue [13,14].

The ribonucleoprotein complex (RNP) of IAV consists of viral genomic RNA segments, multiple nucleoprotein (NP) subunits, and a polymerase complex, which is responsible for the transcription and replication of IAV genome in host cells [15,16]. Compared to the envelope proteins, NP and the polymerase complex are more evolutionarily conserved and are therefore considered the ideal targets for anti-IAV drug development [17,18,19]. Recently, Amorim et al. [20] identified a small-molecule compound Nucleozin, which can prevent the assembly and nuclear import of vRNP through inducing the aggregation of NP. Huang et al. [21] demonstrated that the compound ZBMD-1 can inhibit the nuclear export of NP so as to reduce virus multiplication. Yang et al. [22] discovered that a novel NP inhibitor FA-6005 may target the I41 domain of NP to disrupt the intracellular transport of vRNP during different stages. In addition, Zhang et al. found that a marine fungus derived compound QLA can block the assembly and nuclear export of NP to inhibit IAV replication both in vitro and in vivo [23]. Thus, the NP inhibitors have great potentials to be developed into novel anti-IAV agents in the future.

Mycophenolic Acid (MPA) is a non-competitive, reversible inhibitor of inosine-5′-dehydrogenase with diverse pharmacological activities. Mycophenolic Acid Methyl Ester (MAE), derived from the marine fungus *Phaeosphaeria spartinae*, is a methylated derivative of mycophenolic acid [24]. Both share a benzofuran ring structure, with the carboxylic acid group as the key active site of the molecule. MPA has poor water solubility, whereas in MAE, the carboxylic acid group is replaced by a methyl ester (-COOCH_3_), enhancing lipophilicity and metabolic stability [25]. While MPA and its derivatives exhibit antibacterial [26], immunosuppressive [27], and anticancer [28] effects, MAE’s specific bioactivity of MAE remains largely unexplored. Owing to its marine origin, MAE may possess distinct mechanisms and activities, presenting opportunities for novel drug development. Thus, MAE from marine fungi shows potential as a multifunctional lead compound.

Our previous studies found that the secondary metabolite mycophenolic acid methyl ester (MAE) from the marine fungus *Phaeosphaeria spartinae* exhibited strong anti-IAV activity both in vitro and in vivo, demonstrating low-toxicity broad-spectrum efficacy against IAV and may exert its anti-IAV effects by interfering with the activation of Akt-mTOR-S6K pathway [29]. However, the direct inhibition mechanism of MAE on virus replication needs to be further investigated. In this study, we found that MAE can also interact with virus NP protein to block the nuclear import of virus RNP, and may affect vRNP assembly by blocking NP oligomerization and its binding to PB2 protein. In summary, our findings elucidate the novel antiviral mechanism of MAE, which can provide a reference for the development of marine derived anti-IAV drugs targeting NP protein.

## 2. Results

### 2.1. Screening of Marine Derived Compounds Targeting Virus RNP Complex

**Figure 1:**
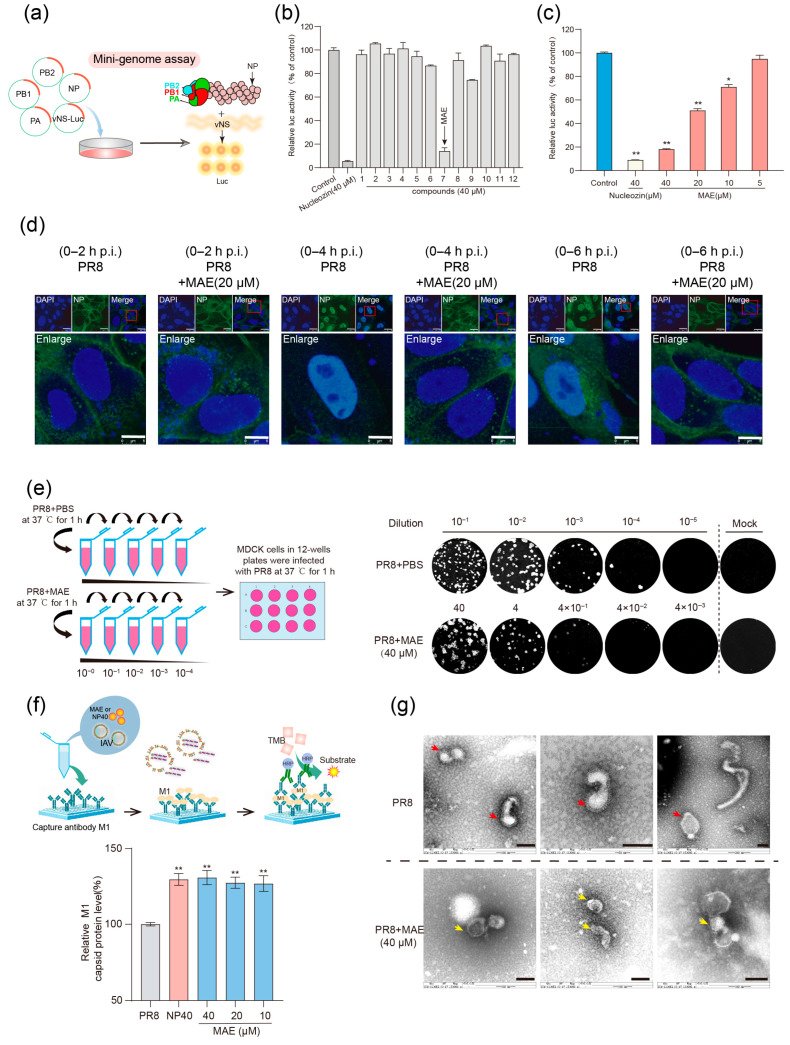
Screening of Marine Compounds Targeting RNP. (**a**) Schematic of the minigenome assay. (**b**) 293T cells were transfected with PA, PB1, PB2, NP, and VNS-Luc plasmids, and the inhibitory activity on vRNP assembly was evaluated using luciferase activity 24 h after the addition of compounds. (**c**) 293T cells were transfected with PA, PB1, PB2, NP, and VNS-Luc plasmids, and the inhibitory activity on vRNP assembly was evaluated using luciferase activity 24 h after the addition of Nucleozin or MAE. * *p* < 0.05, ** *p* < 0.01 vs. Virus Control. (**d**) PR8 (MOI = 1.0) infected MDCK cells with or without MAE (20 μM), the cells were fixed at different times, and the localization of NP in the cells was observed by indirect immunofluorescence. The scale bar represents 25 or 8 μm. (**e**) Virus was incubated with MAE (40 μM) or PBS at 37 °C for 1 h, followed by a 10-fold gradient dilution and infection of MDCK cells for viral titer detection using a phage plaque assay. (**f**) Schematic representation of the Sandwich ELISA. Virus suspension with or without MAE (10–40 μM) was added to enzyme-linked plates coated with M1 antibody, followed by sequential addition of M1 antibody and HRP antibody, and finally detected using TMB. Values are expressed as mean ± S.D. (*n* = 3). ** *p* < 0.01, vs. virus control group. (**g**) Virus suspension with or without MAE (10–40 μM) was dropped onto carbon-coated copper grids, stained with 1% phosphotungstic acid for 4 min, and finally observed using transmission electron microscopy (JEM-1200-EX, Japan Electron Optics Laboratory Co., Ltd., Akishima, Tokyo, Japan). The scale bars represent 100 nm.

The influenza virus RNP complex is an ideal target for the development of antiviral drugs. The minigenome assay is an in vitro system that simulates the assembly and function of virus vRNP complex (Figure 1a), with reconstitution of viral vRNP by transfecting the plasmids encoding virus RNA polymerase complex (PB1, PB2, PA), NP, and vRNA reporter (vNS-Luc). In this study, the minigenome assay was used to screen a small-molecule marine compound library for compounds targeting RNP complex (Table S1). As shown in Figure 1b, among them, compound 7 (MAE) exhibited strong inhibition effect on vRNA synthesis, comparable to that of the reported NP inhibitor Nucleozin. Subsequently, we further found that MAE (10–40 μM) can significantly reduce the luciferase activity of vRNA reporter in a dose-dependent manner compared to the non-treated control group (*p* < 0.05) (Figure 1c). Thus, the results indicate that MAE may affect the function or assembly of vRNP.

To further investigate the influence of MAE on the intracellular localization of the vRNP complex, we detected the NP localization at different time points after virus infection (2 h, 4 h, 6 h p.i.) using an immunofluorescence assay. As shown in Figure 1d, at 2 h p.i., the green fluorescence of NP was detected in the cytoplasm of cells with or without MAE (20 μM) treatment. At 4 h p.i., NP was primarily located in the nucleus of non-treated cells; however, in MAE-treated cells, NP remained mainly in the cytoplasm with minimal nuclear distribution. Similarly, at 6 h p.i., the fluorescence of NP was slightly weak but still localized in the cytoplasm, indicating that MAE can effectively prevent the nuclear entry of viral NP or vRNP (Figure 1d). Besides that, our previous study reported that MAE may exert anti-IAV effects by pre-treating the virus but without interaction with the surface HA and NA proteins [29]. Herein, the results of virucidal assay showed that MAE may also directly inactivate virus particles before infection (Figure 1e), consistent with our previous studies [29].

We conducted a sandwich ELISA experiment (Figure 1f) to investigate whether MAE disrupts the viral envelope. When MAE was administered at 10–40 μM, significant levels of M1 protein within the viral envelope were detected (Figure 1f). At 40 μM MAE, the M1 protein level exceeded that of the NP40-treated positive control, indicating that MAE causes M1 protein release by disrupting the viral envelope. We incubated MAE with viral particles (37 °C, 1 h), adsorbed onto carbon-coated copper grids, stained with 1% phosphotungstic acid, and observed using transmission electron microscopy. The results showed an intact envelope structure in untreated IAV (red arrows, Figure 1g), while MAE-treated IAV showed a ruptured envelope (yellow arrows), confirming that MAE directly inactivates IAV through envelope disruption.

Taken together, MAE not only disrupts the viral envelope to hinder IAV entry, but we also speculate that MAE’s direct inhibitory effect of MAE on viral replication within host cells may stem from its regulation of the function or assembly of the RNP complex.

### 2.2. Compound MAE May Block IAV Infection Through Direct Interaction with NP Protein

**Figure 2:**
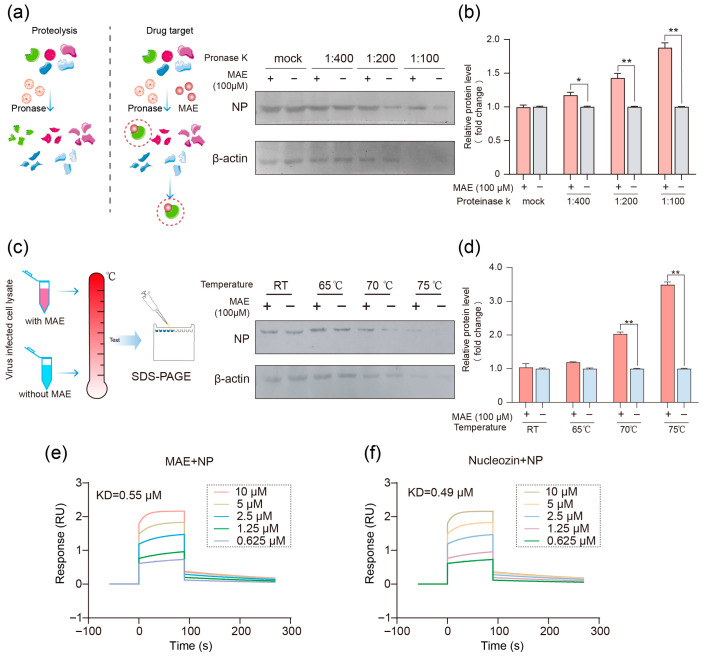
Identification of Marine compound MAE-targeted NP. (**a**) MAE was added or not added to the lysate of PR8-infected MDCK cells, incubated overnight at 4 °C, and different proportions of protease K (5–20 ug/mL) were added. The protective effect of viral protein was detected by Western blot. (**b**) For the quantitative analysis of (**a**), the quantitative software Image J 1.53 was used. * *p* < 0.05, ** *p* < 0.01 vs. Virus Control. (**c**) MAE was either added or not added to the lysate of PR8-infected MDCK cells and incubated overnight at 4 °C, followed by heat treatment at different temperatures for 3–5 min. The protective effect on viral proteins was examined using Western blotting. (**d**) For the quantitative analysis of (**c**), the quantitative software Image J 1.53 was used. ** *p* < 0.01 vs. Virus Control. (**e**,**f**) To assess real-time binding of MAE (**e**) or Nucleozin (**f**) to the NP on CM5 chips, MAE (0.625–10 μM) or Nucleozin (0.625–10 μM) was flowed over the biosensor chip surface. The sensogram for binding interactions was recorded in real time, and the changes in mass due to the binding response were recorded as resonance units (RU).

We further investigated whether NP is a target of MAE by using a drug affinity responsive target stability assay (DARTS) (Figure 2a). In brief, IAV-infected MDCK cell lysates were digested with proteinase K in the presence or absence of MAE (100 μM), then the amount of virus NP protein in cell lysates was detected by Western blot. As shown in Figure 2a,b, virus NP protein can be obviously protected by protease digestion in the extracts of MAE-treated cells especially under proteinase K treatment at 1:100 dilution (20 ug/mL), suggesting that MAE may interact with virus NP protein in IAV infected MDCK cells. Simultaneously, we performed a Cellular Thermal Shift assay (CETSA) to determine whether virus NP protein was targeted by MAE. The results showed that MAE (100 μM) treatment significantly increased the stability of the virus NP protein with increasing temperatures, especially at 70 °C and 75 °C, suggesting that the NP protein may be a target of MAE (Figure 2c,d).

Since the results of DARTS and CETSA experiments indicated that MAE had protective effect on virus NP protein, we further explore the direct interaction between MAE and NP by conducting a surface plasmon resonance (SPR) assay with PR8 virus NP. As shown in Figure 2e, the data revealed a marked binding of MAE (0.625–10 μM) to NP in a dose-dependent manner with a KD equivalent to about 0.55 μM, implicating a high affinity of MAE for NP. The binding of nucleozin to NP was confirmed by SPR assay with the KD value of 0.49 μM, comparable to that of MAE (Figure 2f). Therefore, MAE may block IAV infection through direct interaction with virus NP protein.

### 2.3. MAE May Exert Its Inhibition Effects by Binding to Valine 343 of NP

**Figure 3:**
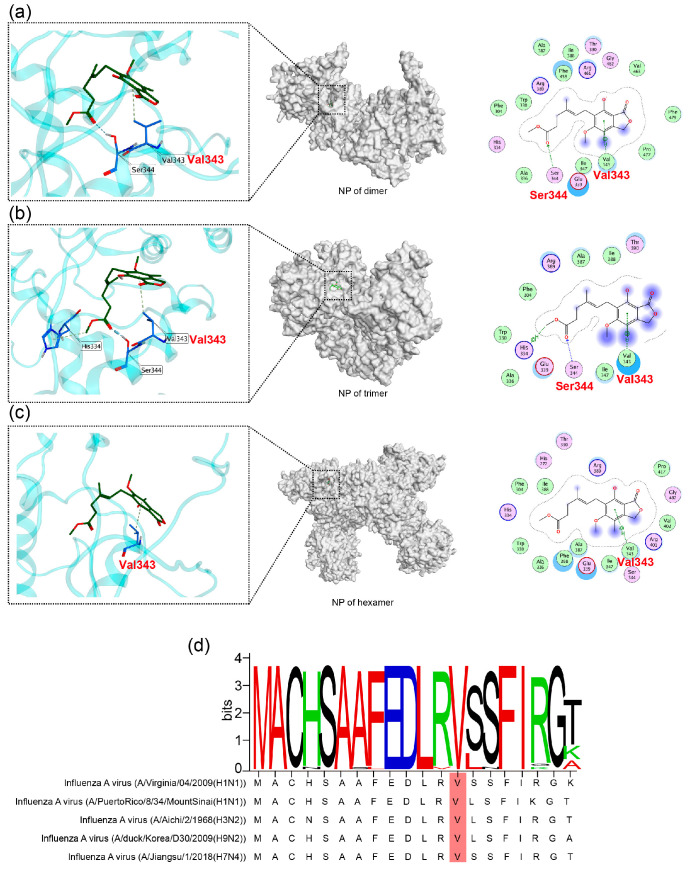
Marine compound MAE may bind to valine at position 343 of the NP. (**a**–**c**) Molecular docking of MAE and different morphologies of NP (NP of dimer PDB: 4DYN (**a**); NP of trimer PDB:3ZDP (**b**); NP of hexamer PDB:2YMN (**c**)) was carried out using MOE-2022.02 software to obtain possible action sites (Val343). (**d**) Thirty NP sequences of different IAV subtypes were randomly downloaded from the National Library of Medicine National Center for Biotechnology Information (www.ncbi.nlm.nih.gov, 1 September 2025) gene database for a conservation analysis (The figure shows the amino acid sequences of five of them).

To further examine how MAE inhibits NP function, we conducted the molecular docking analysis to predict the potential binding sites of MAE to NP. By using the virtual docking, we predicted the binding modes of MAE with NP dimers (Figure 3a), NP trimers (Figure 3b), or NP hexamers (Figure 3c). The results showed that the core ring of MAE could form π-H bonds near Val343, Ser344, Glu339, and Ile347 of NP in various forms. Notably, MAE can bind to Val343 of NP in all forms, with the strongest binding affinity (docking score: −8.68). We attempted to select the NP-resistant viral strains of MAE to verify the biding sites using resistance screening. However, we were unable to obtain any MAE-resistant viral strains, and valine at position 343 of NP did not mutate, suggesting that this binding site (V343) may have high conservation.

To further explore the conservation level of Val343 in NP, we randomly compared the amino acid sequences surrounding Val343 of the NP (MACHSAAFEDLRVSSFIRGK) from 30 virus strains derived from different species. As shown in Figure 3d and Figure S1, the results showed that the ‘MACHSAAFEDLRVSSFIRGK’ sequence of NP is highly conserved in different virus strains, especially for Val343, suggesting that valine at position 343 of virus NP truly has high conservation and is unlikely to develop resistance. In summary, MAE may exert its effects by binding to Val343 of NP, which is a highly conserved site.

### 2.4. MAE Inhibits the Nuclear Entry of NP by Interfering with Its Binding to Importin α

**Figure 4:**
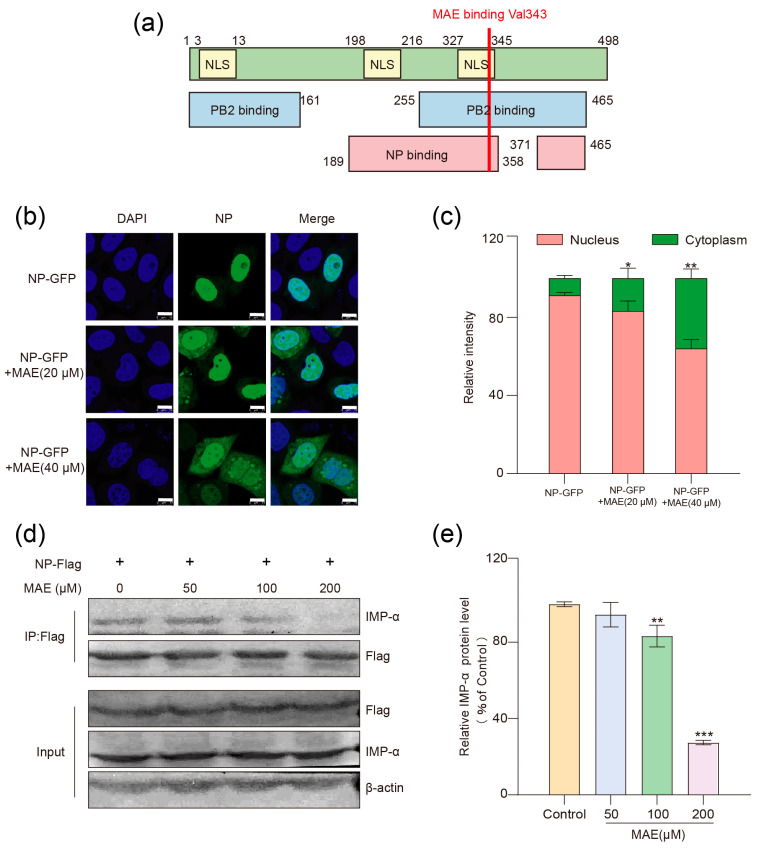
Marine compound MAE influences the entry motion of NP. (**a**) Schematic diagram of the structure of NP. (**b**) NP were inserted into the plasmid pEGFP-C1 to construct GFP chimeric proteins. After transfection with plasmids for 16 h, MDCK cells were treated with MAE (20–40 μM) for 3 h, and the localization of GFP chimeric proteins was detected using confocal microscopy. The scale bar represents 10 μm. (**c**) The relative distribution proportion was analyzed using ImageJ 1.53 software. (**d**,**e**) Lysates from 293T cells transfected or not transfected with the NP-Flag plasmid were incubated with anti-Flag magnetic beads, with MAE (50–200 μM) added to some experimental groups. The binding of IMP-α to NP beads was detected by Western blotting, and the ratio of bound protein to total protein was quantified by immunoblotting (**e**). * *p* < 0.05, ** *p* < 0.01, *** *p* < 0.001.

The NP protein consists of 498 amino acids and can be divided into three functional regions: the nuclear localization signal region (NLS), NP oligomerization region (NP binding), and PB2 binding region (PB2 binding) [30]. The above results of molecular docking showed that MAE may bind to valine 343 of NP to exert its effect. Valine 343 is located within the third nuclear localization sequence (NLS) of NP, the C-terminal PB2 binding region, and the NP oligomerization region (Figure 4a). Thus, we further explored the influence of MAE on the nuclear import and dimerization of NP in order to verify this interaction site.

To further determine whether MAE can exert its effect by blocking the nuclear localization signal of NP, we performed localization of NP-GFP chimeric proteins with or without MAE treatment. In brief, after transfection of plasmids encoding NP-GFP chimeric proteins for 16 h, MAE (20, 40 μM) was added to MDCK cells and incubated for 3 h before observation by confocal microscopy. As shown in Figure 4a,c, in the control group without MAE treatment, most NP were found inside the nucleus; however, MAE (20, 40 μM) treatment significantly increased the fluorescence of NP-GFP in cytoplasm in a dose-dependent manner (*p* < 0.05), indicating that MAE may inhibit the nuclear localization of NP, thereby affecting the nuclear import of viral vRNP.

The nuclear import of vRNP is a multi-step active transport process, which mainly dependents on the interaction between the NLS of NP and the host nuclear transport protein (importin α, IMP-α) [31,32]. Since MAE can block the nuclear localization of NP, we further explored whether MAE can affect the interaction between NP and IMP-α (IMP-α1/KPNA2) by using a pull-down assay. As shown in Figure 4d,e, IMP-α proteins in the cell lysate could be detected on the beads coupled to NP-Flag protein, indicating a direct interaction between NP and IMP-α. However, MAE (100, 200 μM) treatment significantly reduced the binding of IMP-α to NP-Flag beads in a concentration-dependent manner (*p* < 0.01), suggesting that MAE may impede the nuclear import of viral vRNP by blocking NP-IMP-α interaction.

### 2.5. MAE Can Also Bind NP to Block the Assembly of vRNP

**Figure 5:**
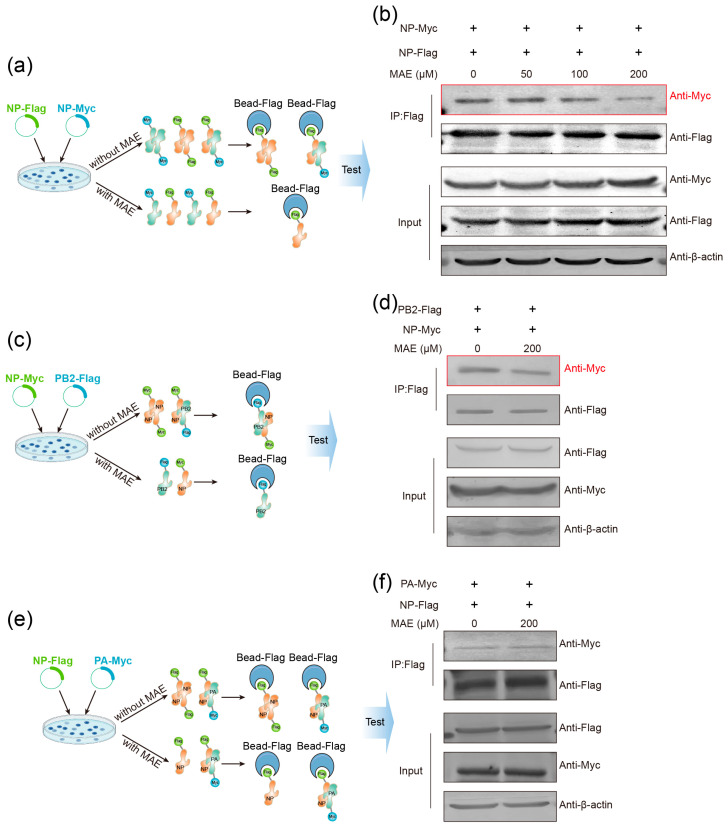
Marine compound MAE affects NP-NP oligomerization and NP-PB2 binding. (**a**,**b**) HEK293T cells were transfected with NP-Flag and NP-Myc, respectively, and the cell lysates were collected and mixed according to the corresponding groups. Different concentrations of MAE were added, incubated at 4 °C overnight, and detected using Western blotting. (**c**,**d**) HEK293T cells were transfected with NP-Myc and PB2-Flag, and the cell lysates were collected and mixed according to the corresponding groups. At the same time, MAE was added, incubated at 4 °C overnight, and detected using Western blotting. (**e**,**f**) HEK293T cells were transfected with NP-Flag and PA-Myc, and the cell lysates were collected and mixed according to the corresponding groups. At the same time, MAE was added, incubated at 4 °C overnight, and detected using Western blotting.

NP participates in the assembly of vRNP as an essential vRNP component. The above results of minigenome assay (Figure 1c) suggested that MAE may affect the assembly of vRNP. Thus, we further examined whether MAE can influence vRNP assembly by affecting NP–polymerase complex interactions. The formation of vRNP requires both NP oligomerization and interaction of NP with the PB2 and PA subunits of the IAV polymerase complex [33,34]. Therefore, we first investigated whether MAE affects NP oligomerization by using the Co-IP assay (Figure 5a,b). The results showed that with the increase in MAE (50–200 μM), the interaction between NP-Flag and NP-Myc was significantly inhibited in a dose-dependent manner (Figure 5b), demonstrating that MAE can block the oligomerization of NP.

Furthermore, we also performed a Co-IP assay to determine whether MAE can affect vRNP assembly by interfering with NP-polymerase interaction (Figure 5c–f). As shown in Figure 5c–f, MAE (200 μM) treatment markedly reduced the binding amount of NP to PB2 (Figure 5d) rather than PA (Figure 5f), suggesting that MAE may affect vRNP assembly via blocking the NP-PB2 interaction rather than NP-PA interaction. In conclusion, MAE may exert its anti-IAV effects by interfering with the NP nuclear localization, NP oligomerization, and vRNP assembly, consistent with the results of molecular docking studies (Figure 3).

## 3. Discussion

Over the past century, global pandemics caused by influenza A viruses have continued to threaten public health, prompting ongoing advancements in strategies for influenza virus prevention and control [35,36]. As the development of terrestrial drug resources becomes increasingly saturated, marine natural products have emerged as a new frontier in antiviral drug discovery owing to their unique chemical diversity and bioactivity [37,38,39]. The RNP of the influenza A virus is an ideal target for developing new anti-IAV drugs. Through mini-genome assay, we screened a small marine compound library and discovered that Mycophenolic Acid Methyl Ester, a secondary metabolite from the marine alga-associated fungus *Phaeosphaeria spartinae*, can effectively inhibit the formation of RNP in vitro.

In this study, we first analyzed the target and molecular mechanisms of action of MAE during the early stages of viral infection. Indirect immunofluorescence assays showed that MAE significantly inhibited NP-mediated nuclear localization of vRNP, suggesting that it may function by interfering with NP. To test this hypothesis, we used DARTS, thermal shift, and molecular docking techniques and confirmed that MAE specifically binds to the Val 343 site of the NP. This site is located in a key functional region of the NP, involving the nuclear localization signal, oligomerization domain, and interaction area between NP and PB2. Based on these findings, we systematically studied the regulatory effect of MAE on NP function. Co-immunoprecipitation experiments showed that MAE treatment significantly reduced the interaction between NP and the nuclear transport factor importin-α, resulting in impaired nuclear import of vRNP. In addition, MAE inhibited NP oligomerization and NP-PB2 complex formation in a dose-dependent manner. Based on previous research findings [29], MAE has demonstrated good anti-IAV activity, both in vitro and in vivo. We further investigated the direct interaction between MAE and the virus. Viral inactivation assays and sandwich ELISA experiments showed that MAE directly interacts with the virus and disrupts the viral envelope. This direct inactivation effect suggests that MAE has the potential to be developed as a disinfectant or spray in the future.

In current studies related to NP inhibitors, Nucleozin [20] prevents vRNP assembly and nuclear import by inducing NP aggregation. In contrast, this study found that MAE specifically binds to the highly conserved Val343 site of NP, rather than inducing NP aggregation, thereby inhibiting the NP-IMP-α interaction (blocking nuclear import), NP oligomerization, and NP-PB2 binding (disrupting vRNP assembly). This mode of action is more precise, and the conserved nature of Val343 suggests that MAE is less likely to induce resistance. ZBMD-1 [21] reduces viral replication by inhibiting NP nuclear export, whereas MAE’s primary mechanism of action of MAE is to block NP nuclear import, thus targeting different stages of vRNP nuclear transport. Additionally, MAE also has the ability to directly inactivate viral particles (Figure 1e–g). Next, we will focus on in vivo target validation experiments for MAE. Using a reverse genetics system to package NP viruses with a Val343 site mutation, we will verify the anti-IAV efficacy of MAE against mutant strains in vivo. Simultaneously, we will assess the pharmacokinetic parameters of MAE in mice (such as blood drug concentration, half-life, and bioavailability) and evaluate its toxicity in major organs (liver and kidneys) to determine its potential for clinical application.

Notably, Val343 is located at the intersection of three key functional regions of NP: the C-terminal nuclear localization signal (which mediates nuclear import), NP oligomerization domain (which supports vRNP assembly), and PB2 binding site (which promotes integration of the polymerase complex) (Figure 4a). This explains why the binding of MAE to Val343 can simultaneously inhibit NP-IMP-α interaction (Figure 4d,e), NP oligomerization (Figure 5a,b), and NP-PB2 binding (Figure 5d), which are three processes that are critical for vRNP function. In addition, sequence analysis of 30 IAV subtypes (Figure 3d and Figure S1) showed that Val343 is completely conserved, even in highly pathogenic strains such as H5N1 and H7N9, suggesting that MAE may avoid drug resistance caused by target mutations, which is a major limitation of current anti-IAV drugs such as oseltamivir and baloxavir.

As a core structural protein, NP is indispensable in the processes of viral RNA packaging, particle assembly, host cell entry, and replication/transcription of the influenza virus. Moreover, NP possess host immune regulatory functions. Consistent with previous conclusions, combining prior research with the results of this study, we speculate that MAE promotes the production of type I interferons by activating the NP-mediated Akt-mTOR-S6K signaling pathway, thereby inhibiting viral replication. In this study, we found that MAE can block NP-mediated vRNP assembly and nuclear import by specifically binding to Val 343 of the NP. These findings enhance our understanding of the anti-IAV mechanisms of MAE. To date, no anti-IAV drugs targeting NP have been approved for use. Therefore, this study provides valuable insights for the development of NP-targeting anti-IAV drugs. Furthermore, considering the multi-pharmacological activities of MAE, its potential applications in other viral infections and inflammatory diseases merit further investigation.

## 4. Materials and Methods

### 4.1. Compounds and Reagents

Mycophenolic Acid Methyl Ester (MAE) is a compound found in the marine fungus *Phaeosphaeria spartinae*. Methyl mycophenolate is the methyl ester of mycophenolic acid. MAE (purity > 99%) was purchased from Topscience (Shanghai, China). Rabbit monoclonal anti-PA antibody was purchased from GeneTex (Irvine, CA, USA). Rabbit monoclonal anti-HA and anti-NA antibodies were purchased from SinoBiological (Beijing, China). Mouse monoclonal anti-Flag and β-actin antibodies were purchased from Cell Signaling Technology (Danvers, MA, USA). The SDS-PAGE Gel Preparation Kit was purchased from Beyotime (Shanghai, China).

### 4.2. Cell Culture and Viral Infection

MDCK cells from Cell Bank of CAS (Shanghai, China). PR8 (A/Puerto Rico/8/34 [H1N1]; PR/8) from American Type Culture Collection (Manassas, VA, USA). MDCK cells were chosen as the cell model for IAV infection experiments due to their high susceptibility. MDCK cells are the gold standard for IAV plaque assays and virus titer determination, forming clear and reproducible plaques for quantitative analysis. Unlike Vero cells, MDCK cells have low innate antiviral responses, ensuring accurate assessment of antiviral activity, making them ideal for studying IAV infections. MDCK cells were grown in Dulbecco’s Modified Eagle’s medium (DMEM) supplemented with 10% fetal bovine serum (FBS), 100 U/mL penicillin, and 100 μg/mL streptomycin. (Human Embryonic Kidney 293T cells) HEK293T cells were maintained in DMEM containing 10% FBS, 100 U/mL penicillin, and 100 μg/mL of streptomycin. Influenza virus (A/Puerto Rico/8/34 [H1N1]; PR/8) was propagated in 9-day-old embryonated eggs for 3 days at 36.5 °C.

### 4.3. Mini-Genome Assay

HEK293T cells were chosen for assays because of their high transfection efficiency (~80–90% with PEI), which is essential because the minigenome assay requires the simultaneous expression of five plasmid components (PB1, PB2, PA, NP, and vNS-Luc) to form functional vRNP; low efficiency would reduce assay sensitivity. MDCK cells, with low transfection efficiency (~20–30%), are unsuitable for these assays. HEK293T cells were evenly seeded in a 6-well plate and transfected when they reached 70–80% confluency. Plasmids were transfected using PEI in the ratio NP:PA:PB1:PB2:VNS-Luc = 1:1:1:1:2. After transfection for 6 h, the medium was replaced with complete DMEM containing MAE. A positive control group treated with Nucleozin was also included. After 24 h of incubation, the cells were lysed, and the lysates were transferred to a 96-well white plate. Fluorescence values were measured using a Firefly Luciferase Reporter Gene Assay Kit from the Beyotime Biotechnology (Shanghai, China).

### 4.4. Surface Plasmon Resonance (SPR) Assay

The influenza virus NP was coupled to a CM5 chip and activated with NHS/EDC. The experiment was conducted in a PBST solution containing 10% DMSO. The compound was serially diluted and passed over the chip surface at a flow rate of 20 μL/min, with the interaction set for 60 s and dissociation for 2 min. Kinetic and affinity fitting were used to obtain KD values. The binding capacity was evaluated using sensorgrams and KD values, with nucleozin targeting the NP as a positive control.

### 4.5. Drug Affinity Responsive Target Stability (DARTS) Assay

PR8 influenza virus (MOI = 0.1) was used to infect MDCK cells, which were then incubated at 37 °C and 5% CO_2_ for 1 h. After removing the inoculum, viral replication was allowed to proceed for 16 h under the same conditions. At the end of the culture cycle, 100 μL of MPER lysis buffer was added, and intracellular proteins were released during a 20 min incubation on ice. Protein lysate was then collected. The protein lysate was mixed with 1 × TNC buffer, with or without MAE, and incubated overnight at 4 °C with continuous rotation at 15 rpm to ensure sufficient binding between the drug and the target. Proteinase K (0.5 μg/mL, 1 μg/mL, or 2 μg/mL) was then added or omitted, and after a 10 min reaction at 37 °C, 1 μL of EDTA was immediately added and vortexed thoroughly to terminate the reaction. Detection was performed using Western blotting.

### 4.6. Thermal Shift Assay (TSA)

PR8 influenza virus (MOI = 0.1) was used to infect MDCK cells, which were then placed in a 37 °C, 5% CO_2_ incubator for 1 h. After removing the inoculum, viral replication was allowed to proceed under the same conditions for 16 h. At the end of the culture cycle, 100 μL of NP40 lysis buffer was added, and intracellular proteins were released during a 20 min incubation on ice. The protein lysate was collected. 1 × TNC buffer, with or without MAE, was mixed with the protein lysate and incubated overnight at 4 °C with continuous rotation at 15 rpm to ensure sufficient binding between the drug and the target. The mixture was then heated for 3 min at 65 °C, 70 °C, and 75 °C, respectively (the specific temperatures should be determined by a preliminary experiment, generally within the range of 40 °C to 80 °C). After heating, the samples were immediately centrifuged for 1 min, and the supernatant was collected. Western blotting was used for detection.

### 4.7. Computer-Aided Virtual Docking Experiment

MOE software was used to dock different full-length NP (PDB: 4DYN, 3ZDP,2YMN) with MAE (CAS: 31858-66-9). First, small-molecule compounds and proteins were rapidly pre-processed. The full-length proteins were then docked with small molecules. The docking mode and parameters were adjusted, with semi-flexible docking (protein fixed, small molecule conformation allowed to change) used by default. The docking program was then initiated on the selected compounds. Upon completion, conformations with the highest binding scores or the most interactions were selected for visualization and analysis, and the strongest affinity-binding sites were identified and analyzed.

### 4.8. Pull Down Assay

HEK293T cells were evenly seeded in a 6-well plate and transfected when they reached 70–80% confluency. PEI was used to transfect 4 μg of NP-Flag and NP-Myc (or PA, PB2) plasmids. 6 h after transfection, the medium was replaced with a medium containing or lacking MAE. After incubation for 24–36 h, the cells were lysed and collected. The cell lysate was incubated overnight with Bead-Flag at 4 °C and 15 rpm. After incubation, the supernatant was discarded, and the beads were washed with 1× PBST solution. Detection was performed using the Western blot assay.

### 4.9. Virucidal Assay

PR8 virus was mixed with MAE (40 μM) or PBS and incubated at 37 °C for 1 h. Subsequently, the mixture was subjected to a 10-fold serial dilution (10^−1^–10^−5^) and incubated with MDCK cells at 4 °C for 1 h. A plaque assay was performed to detect viral titers.

### 4.10. Statistical Analysis

All data represent at least three independent experiments per group. Data are expressed as mean ± standard deviation (SD). Statistical significance was calculated using SPSS 10.0 software with one-way ANOVA and Tukey’s test or the non-parametric Wilcoxon rank-sum test, with a *p*-value < 0.05 considered significant.

## 5. Conclusions

In summary, this study identified mycophenolic acid methyl ester (MAE), a secondary metabolite from the marine algal-associated fungus *Phaeosphaeria spartinae*, as a potent nucleoprotein (NP) inhibitor of the influenza A virus (IAV). Key findings include: (i) MAE directly targets IAV NP by binding to the highly conserved Val343 site (docking score: −8.68, KD: ~0.55 μM), as verified by DARTS, CETSA, and SPR assays. (ii) MAE inhibits IAV replication through three synergistic mechanisms: blocking vRNP nuclear import by disrupting NP-IMP-α interaction; inhibiting vRNP assembly by reducing NP oligomerization and NP-PB2 binding; and directly inactivating viral particles by damaging the viral envelope. (iii) Val343 of NP is conserved across 30 IAV subtypes (including H1N1, H3N2, H5N1, and H7N9), suggesting that MAE has the potential for broad-spectrum anti-IAV activity and a low risk of drug resistance.

These findings not only clarify MAE’s dual mode of action of MAE (targeting viral proteins and disrupting the envelope) but also establish Val343 as a novel therapeutic target for IAV. In future research, in vivo validation of MAE’s efficacy and safety in animal models, optimization of MAE derivatives to enhance potency, and testing against diverse IAV subtypes will be critical to advance MAE toward clinical development as a marine-derived anti-IAV drug.

## Data Availability

The data presented in this study are available on request from the corresponding authors.

## Supplementary Materials

The following supporting information can be downloaded at: https://www.mdpi.com/article/10.3390/md23110413/s1, Figure S1: The Val343 of NP in IAV is highly conserved. Thirty NP sequences of different IAV subtypes were randomly downloaded from the National Library of Medicine National Center for Biotechnology Information (www.ncbi.nlm.nih.gov, 1 September 2025) gene database for a conservation analysis; Table S1: The marine derived small molecules screened in this study; Table S2: Antiviral activity and binding affinity of MAE and Nucleozin against influenza viruses.

## Abbreviations

The following abbreviations are used in this manuscript:

EDC	1-Ethyl-3-(3-Dimethylaminopropyl) Carbodiimide Hydrochloride
FBS	Fetal Bovine Serum
IAV	Influenza A Virus
IMP-α	Importin-α
KD	Binding Affinity (Kd = Kd(Dissociation Rate Constant)/Ka (Association Rate Constant))
MAE	Mycophenolic Acid Methyl Ester
MOE	Molecular Operating Environment
NHS	N-Hydroxysuccinimide
NLS	Nuclear Localization Signal Region
NP	Nucleoprotein
PA	Polymerase Acid Protein
PB1	Polymerase Basic Protein 1
PB2	Polymerase Basic Protein 2
PDB	The Brookhaven Protein Data Bank
SPR	Surface Plasmon Resonance
TNC	Tris-Nacl-Cacl_2_
vRNP	Viral Ribonucleoprotein Complex
