# Supplementary material for: Identifying a Marine-Derived Small-Molecule Nucleoprotein Inhibitor Against Influenza A Virus
Source: Mar Drugs. 2025 Oct 23;23(11):413. doi: 10.3390/md23110413 (PMC12653688; doi:10.3390/md23110413)
Supplement: Supplementary file 1 [file marinedrugs-23-00413-s001.zip › marinedrugs-3912091-supplementary.pdf]

Supplementary Materials for

Identifying a Marine-Derived Small-Molecule Nucleoprotein Inhibitor Against Influenza A Virus

Zihan Wang †, Yang Zhang †,*, Shangjie Xu, Lishan Sun, Hongwei Zhao and Wei Wang *

Key Laboratory of Marine Drugs, Chinese Ministry of Education; School of Medicine and Pharmacy, Ocean University of China, 5 Yushan Road, Qingdao, 26003, China; wzzz0911@163.com (Z.W,); 17630979435@163.com (S.X.); 17806274691@163.com (L.S.); hweizhao2024@163.com (H.Z.)

* Correspondence: zhangyang@ouc.edu.cn (Y.Z.); Tel.: +86-532-8203-1980; wwwakin@ouc.edu.cn (W.W.);Tel.: +86-532-8203-1980.

† These authors contributed equally to this work.

The PDF file includes: Figures S1 and Tables S1 and S2

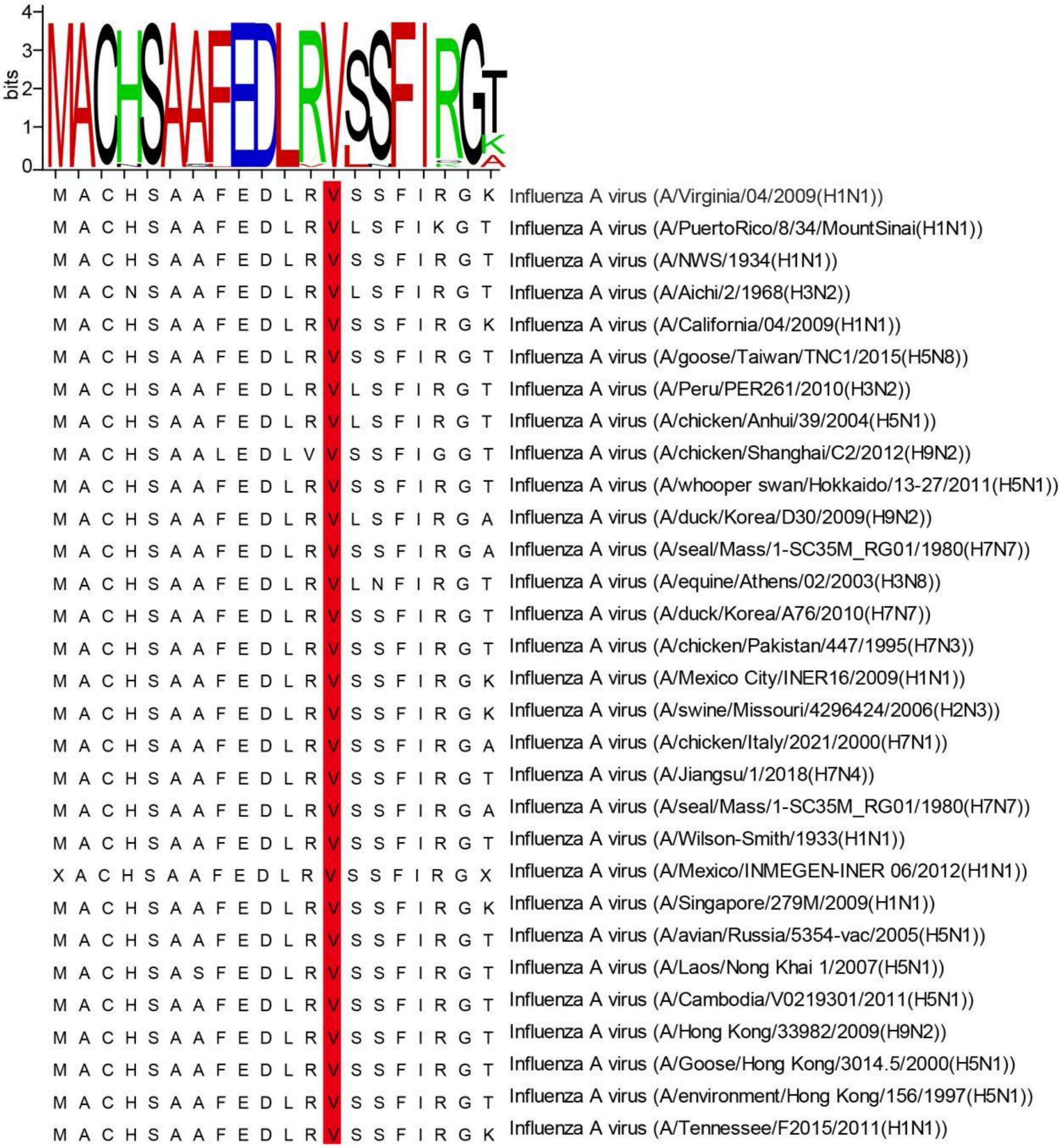

Figure S1 The Val343 of NP in IAV is highly conserved. Thirty NP sequences of different IAV subtypes were randomly downloaded from the National Library of Medicine National Center for Biotechnology Information (www.ncbi.nlm.nih.gov) gene database for a conservation analysis.

Table S1 The marine derived small molecules screened in this study

Number	Compounds	CAS	Structures
compound 1	(S)-(+)-ochracin	1200-93-7	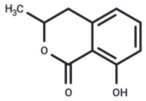
compound 2	6-Hydroxyisatin	116569-08-5	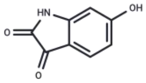
compound 3	6-Bromo-1H-indole-3-acetic acid methyl ester	152213-63-3	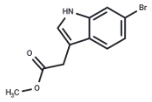
compound 4	1,3-Diphenethylurea	5467-84-5	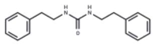
compound 5	N-methyl-1H-indole-2-carboxamide	69808-71-5	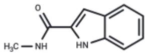
compound 6	1,7-dimethyl-1H-indole-3-carbaldehyde	164353-61-1	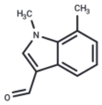
compound 7	Mycophenolic Acid Methyl Ester	31858-66-9	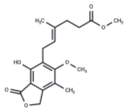
compound 8	4,5-Dibromo-1H-Pyrrole-2-Carboxylic Acid	34649-21-3	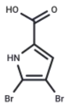
compound 9	Cyclo-Val-Pro-diketopiperazine	5654-87-5	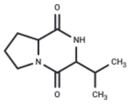
compound 10	Allocholic acid	2464-18-8	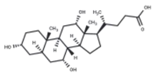
compound 11	2-Chlorobenzene-1,3,5-triol	84743-76-0	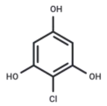
compound 12	Cyclo(Pro-Leu)	5654-86-4	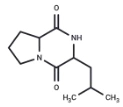

Table S2. Antiviral activity and binding affinity of MAE and Nucleozin against influenza viruses

Compound Name	Virus Subtype	IC_{50} (μM)[a]	Binding Affinity KD (μM)[b]
MAE	A/Puerto Rico/8/1934 (H1N1)	14.3±2.6	0.55
	A/California/04/2009 (H1N1)	3.3±2.4	
	A/Aichi/2/1968 (H3N2)	3.4±2.1	
Nucleozin	A/Puerto Rico/8/1934 (H1N1)	14.2±2.2	0.49
	A/California/04/2009 (H1N1)	4.8±1.8	
	A/Aichi/2/1968 (H3N2)	6.6±1.7	

[a] IC_{50} values were determined by a cytopathic effect (CPE) inhibition assay in Madin-Darby Canine Kidney (MDCK) cells at an MOI of 0.1. Data represent the mean ± SD from three independent experiments.
[b] KD values were determined by Surface Plasmon Resonance (SPR) analysis, measuring the direct interaction with the influenza nucleoprotein (NP).
